# Editorial: Methods in speech and language: 2023

**DOI:** 10.3389/fnhum.2024.1475311

**Published:** 2024-08-19

**Authors:** Anastasios M. Georgiou, Susan Jerger

**Affiliations:** ^1^The Cyprus Rehabilitating Aphasia and Dysphagia Lab, Department of Rehabilitation Sciences, Cyprus University of Technology, Limassol, Cyprus; ^2^The Brain and Neurorehabilitation Lab, Department of Rehabilitation Sciences, Cyprus University of Technology, Limassol, Cyprus; ^3^Undergraduate Honors Program and Child Speech Lab, School of Behavioral and Brain Sciences, The University of Texas at Dallas, Richardson, TX, United States

**Keywords:** dysarthria, event-related potential (ERP), auditory feedback, dysphagia, intensive care, brain artificial intelligence

With rapid technological advancements, speech and language research has expanded significantly over the last two decades, impacting disciplines such as neuroscience, linguistics, computer and cognitive science. Cutting-edge experimental methods have allowed researchers to explore the complexities of speech and language more thoroughly, unraveling new insights and driving both theoretical and practical innovations. Techniques like neuroimaging, electrophysiology, computational modeling and machine learning algorithms have improved our understanding of human communication. Beyond addressing communication disorders, speech and language therapy (SLT) also covers swallowing disorders (aka dysphagia) which often accompany speech and language impairments. Addressing dysphagia is vital for improving individuals' quality of life (QoL) and requires specialized assessment and intervention techniques. The present Research Topic, part of the Methods in Human Neuroscience series, highlights the latest techniques and methods in speech and language research. The six featured articles include original research, detailed protocols, and insightful reviews, all making a substantial impact on the field. Together, these studies offer valuable insights for clinicians and researchers dedicated to improving the lives of individuals with communication disorders and dysphagia.

Dysarthria is a neuromotor disorder characterized by weakness or coordination difficulties in the vocal and respiratory muscles (Enderby, 2013). These challenges produce imprecise articulation, monotonous speech prosody, reduced voice quality, and inadequate respiratory control. Treatment approaches for dysarthria include speech therapy to enhance speech production skills and augmentative therapies, such as gesture use, to improve communication. Specific speech therapy methods include Phonemic Contrast Therapy, which focuses on enhancing articulatory precision and auditory phoneme discrimination, and the Accent Method, which targets improving respiratory control and voice production. The research of Ge et al. innovatively integrated these two methodologies into a more holistic approach for improving speech production in speakers with post-stroke dysarthria. The results demonstrated that this synergistic technique offered significant advantages in optimizing the segmental (speech clarity) and suprasegmental (prosody) aspects of speech production. Research about more effective methods to improve speech production is crucial for enhancing access to education, employment, and socialization opportunities in individuals with dysarthria.

The event-related potentials (ERPs) technique is important for exploring different levels of linguistic structure, including phonetic (Whitten et al., 2020), lexical (Xia and Peng, 2022), syntactic (Deng and Zhu, 2020), and discourse analysis (Rasenberg et al., 2019). Despite many reviews, a comprehensive overview of ERPs research in language processing is lacking. Sun and Luo's study addresses this gap by using Citespace to analyze 3,772 publications from 2002 to 2022, providing insights into historical developments, current trends, and future predictions. Important research areas include sentence and reading comprehension, mismatch negativity, and topics like speech perception, temporal dynamics, and working memory. The study calls for future research on larger linguistic units, ERP component integration, and individual differences to improve the understanding of language processing. This comprehensive analysis broadens our understanding of ERPs applications in linguistic research, guiding future studies in the field.

Auditory feedback is essential for speech production and is central to speech motor control models (Parrell et al., 2019). Over the past 30 years, the real-time altered auditory feedback (AAF) paradigm has become popular for studying auditory feedback control. This technique changes a speaker's speech and feeds it back in approximately real-time. Tang reviewed AAF's development for studying pitch motor control in tonal language speakers, analyzing 18 studies. The findings indicate that tonal language speakers can adapt to pitch perturbations, with response magnitude and latency affected by various factors. Combining AAF with brain stimulation and neuroimaging techniques allows for the exploration of the neural basis of pitch motor control. This review highlights AAF's importance in understanding pitch motor control and its potential applications in speech research and therapy.

Neurolinguistic assessments are crucial but often time-consuming in neurological exams. Open Brain Artificial Intelligence (OBAI) (http://openbrainai.com) aims to improve these processes with advanced AI tools for analyzing spoken and written language. Initially developed by one person (Themistocleous), OBAI now needs a research group for further development and global impact. The platform supports multiple languages and offers models for several linguistic tasks (e.g., audio transcription, translation, and grammar correction). “OBAI Companion” helps clinicians in structuring and summarizing texts, improves functionality, innovation, and the platform's impact, enabling professionals to focus on therapeutic interventions. Themistocleous reviews OBAI's architectures and applications, underscoring its significance in making neurolinguistic assessments more efficient and less resource-intensive.

Dysphagia impacts about 40% of stroke survivors (Balcerak et al., 2022), emphasizing the urgent need for effective treatments. Although several studies have examined the effects of repetitive Transcranial Magnetic Stimulation (rTMS) on post-stroke dysphagia, there is no consensus on the optimal stimulation site and parameters (Li et al., 2022). The umbrella review of Georgiou et al. evaluated the methodological quality of systematic reviews (SRs) and meta-analyses (MAs) on rTMS's effectiveness in managing post-stroke dysphagia. Using AMSTAR 2 (A Measurement Tool to Assess Systematic Reviews) (Shea et al., 2017), the researchers rated two studies as low quality and 17 as critically low, noting high literature overlap. Despite methodological flaws, all MAs found significant evidence supporting rTMS's effectiveness. This research is important as it shows the potential of rTMS in improving post-stroke dysphagia, guiding future studies toward more robust methodologies.

Last but not least, the study of Demetriou and Georgiou illustrate the absence of standardized protocols and evidence-based guidelines for post-extubation dysphagia (PED) screening and assessment in intensive care units (ICUs). Their paper explored current dysphagia screening and assessment methods as well as existing dysphagia protocols in ICUs, emphasizing the need for innovative approaches. Their study urges the establishment of internationally standardized, patient-centered guidelines to improve diagnosis, prevention, and management of PED. Such guidelines would reduce the burden on critically ill patients, ensure consistency across the world, enhance the effectiveness of interventions, and support international educational programs. This step is crucial for improving global PED practices and patient outcomes.

Overall, this editorial illustrates the need for continued innovation and methodological advancements in speech and language research, emphasizing the potential for future studies to improve diagnostic precision, therapeutic outcomes, and QoL for people with communication disorders and dysphagia.
